# Conservation of Olfactory Avoidance in *Drosophila* Species and Identification of Repellents for *Drosophila suzukii*

**DOI:** 10.1038/srep11527

**Published:** 2015-06-22

**Authors:** Christine Krause Pham, Anandasankar Ray

**Affiliations:** 1 Interdepartmental Neuroscience Program, University of California, Riverside, CA 92521.; 2 Entomology Department, University of California, Riverside, CA 92521.

## Abstract

Flying insects use olfaction to navigate towards fruits in complex odor environments with remarkable accuracy. Some fruits change odor profiles substantially during ripening and related species can prefer different stages. In *Drosophila* species attractive odorants have been studied extensively, but little is understood about the role of avoidance pathways. In order to examine the role of the avoidance cue CO_2_ emitted from fruit on behavior of two species with different ripening stage preferences, we investigated the CO_2_-detection pathway in *Drosophila melanogaster* and *Drosophila suzukii*, a harmful pest of fruits. Avoidance to CO_2_ is not conserved in *D. suzukii* suggesting a behavioral adaptation that could facilitate attraction to younger fruit with higher CO_2_ emission levels. We investigated known innate avoidance pathways from five species at different evolutionary distances: *D. melanogaster, D. yakuba, D. suzukii, D. pseudoobscura* and *D. virilis*. Surprisingly, only DEET shows strong repellency across all species, whereas CO_2_, citronellal and ethyl 3-hydroxybutyrate show only limited conservation. These findings guide us to test recently discovered safe DEET substitutes, and we identify one that protects fruits from *D. suzukii* thus providing a new behavioral strategy for controlling agricultural pests.

The two related species, *Drosophila melanogaster* (vinegar fly) and *Drosophila suzukii* (spotted wing *Drosophila*) provide an excellent model to study how changes in olfactory behavior are associated with changes in food preference. Drastic changes occur in the composition of volatiles emitted during fruit maturation, ripening and fermentation, potentially providing different cues for different species. *D. melanogaster* primarily feed and lay eggs on overripe and fermenting fruits often fallen from plants and avoid fruit that is still ripening on the plant. During the ripening process, fruits undergo higher rates of respiration and emit a higher level of CO_2_^1^, which *D. melanogaster* strongly avoid^2^,^3^,^4^,^5^,^6^. As the fruit over-ripens, levels of CO_2_ emission decrease and a concomitant increase in yeast-derived volatiles occur. Volatiles from yeast can attract flies, and there are some (such as hexanol and 2,3-butanedione) that can also inhibit the aversive CO_2_ receptor^7^ (Fig. 1A). Conversely, *D. suzukii* prefer to feed on ripening fruits and have evolved a specialized ovipositor that can tear through the skin of ripe berries to lay eggs^8^,^9^ and their larvae emerge inside causing hundreds of millions of dollars worth of agricultural damage worldwide^10^,^11^.

Here we examine the olfactory determinants underlying these behavioral preferences and our findings can serve not only as an important model to understand adaptations in behavior, but can also be exploited to control insects that are harmful to humans. While attractive odor cues emitted from food play an important role in the differential selection process across species^12^,^13^, here we investigate the less studied changes in behavior towards aversive cues.

## Results

### CO_2_ avoidance behavior is not conserved in all Drosophila species

**Figure 1:**
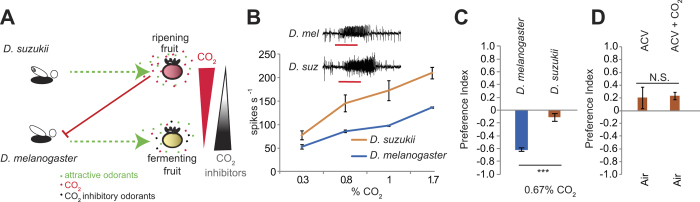
*D. suzukii* do not avoid carbon dioxide like *D. melanogaster* **(A)** Schematic depicting proposed roles of CO_2_ and volatiles emitted from fruit contributing to attraction behavior. *D. melanogaster* and *D. suzukii* are attracted to fermenting and ripe fruits respectively. **(B)** Mean electrophysiological responses of the ab1C neuron to different doses of carbon dioxide. N = 5–6 recordings/concentration. **(C)** Mean preference index of *D. melanogaster* and *D. suzukii* to carbon dioxide (0.67%) in a T-maze assay. N = 11–39 trials, 40 flies/trial, T-test (***P < 0.001). **(D)** Mean preference index of *D. suzukii* to 10% apple cider vinegar in the context of a choice with air or CO_2_ (0.33%) in T-maze assay. N = 4 trials, 30 flies/trial, T-test (P = 0.88).

In order to ask whether the CO_2_-avoidance pathway has been adapted to suit the *D. suzukii* change in behavior, we first investigated their ability to detect CO_2_. The CO_2_ receptor is comprised of two 7-transmembrane proteins Gr21a and Gr63a, which are housed in the ab1C neuron on the *D. melanogaster* antenna. We found that the amino acid sequences of both Gr21a and Gr63a are extremely well conserved in the *D. suzukii* genome (99% and 94%, respectively). In order to test whether the functional expression of the receptors occurs, we used single sensillum electrophysiology on the *D. suzukii* antenna (N = 5–6). Our results indicate that an ab1C-like neuron in *D. suzukii* is present and is in fact more sensitive to CO_2_ than *D. melanogaster* across different concentrations (Fig. 1B). We next tested *D. suzukii* preference for CO_2_ in a T-maze Assay. Surprisingly, *D. suzukii* did not show avoidance to CO_2_ at levels that elicit robust avoidance in *D. melanogaster* (P < 0.001) (Fig. 1C). These results suggest that while *D. suzukii* can detect CO_2_ other changes in processing this sensory information have occurred. Since *D. suzukii* are attracted to ripening fruits which emit CO_2_, we also tested whether CO_2_ can enhance attraction in the presence of food odors such as apple cider vinegar (ACV) as has been reported in *D. melanogaster*^6^. *D. suzukii* showed no significant increase in attraction to ACV in the presence of CO_2_ (P = 0.88) (Fig. 1D). While we do not know the behavioral significance of CO_2_ detection in this species yet, the ability to sense but not avoid CO_2_ may offer a distinct evolutionary advantage since *D. suzukii* feed on ripening fruits that respire and emit CO_2_.

**Figure 2:**
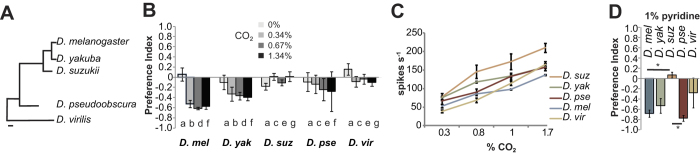
Conservation of the CO_2_ avoidance pathway across various *Drosophila* species **(A)** Phylogenic tree of selected *Drosophila* species (adapted from^32^). **(B)** Mean preference index of the various *Drosophila* species to the indicated doses of carbon dioxide in a T-maze Assay. N = 5–39 trials, 40 flies/trial, error bars = S.E.M. The Holm-Sidak method was used to conduct a pair-wise multiple comparison of species and CO_2_ concentration. There was a significant difference between air and all three CO_2_ concentrations in D*. melanogaster* (P < 0.05). *D. yakuba* avoided CO_2_ at all concentrations, but not significantly. **(C)** Mean electrophysiological responses of the antennal large basiconic CO_2_-sensing neuron to different doses of carbon dioxide across the various species. N = 5–6 recordings/concentration. **(D)** Mean preference index of the *Drosophila* species to 1% pyridine in T-maze Assay. Control arm contains paraffin oil. N = 5–10 trials, 40 flies/trial, error bars = S.E.M. For Kruskal-Wallis One Way Analysis of Variance on Ranks there is a difference in the mean values (P = 0.005). Specifically, in a pair wise multiple comparison procedure (Dunn’s Method), *D. suzukii* respose differs from both *D. melanogaster* and *D. pseudoobscura* (P < 0.05).

Avoidance of CO_2_ is robust in the *D. melanogaster* lab strain and also in two recently caught wild-type strains tested in the T-maze Assay which suggests that repellency is not due to artificial selection in our lab strains (P = 0.01, P = 0.03) (Supplementary Fig. S1). These results pose an interesting question: how conserved is avoidance of CO_2_ in other related *Drosophila* species? In order to answer this question we performed a series of behavioral and electrophysiological experiments using 3 additional *Drosophilid* species (Fig. 2A). Using the T-maze Assay we found that the closely related *D. yakuba* showed avoidance to carbon dioxide, albeit to a lower degree than *D. melanogaster* (Fig. 2B). However, like *D. suzukii*, the more distantly related *D. virilis* did not avoid carbon dioxide and *D. pseudobscura* was only mildly (but not significantly) repelled at the highest concentration tested (Fig. 2B). The Gr21a and Gr63a amino acid sequences are highly conserved across all tested species: *D. yakuba* (100% and 97%), *D. pseudoobscura* (97% and 93%) and *D .virilis* (88% and 85%). We find that a CO_2_-sensitive ab1C-like neuron is present in each of these species from single sensillum recordings (Fig. 2C). These results suggest that detection of CO_2_ is conserved; however, the behavioral changes could likely be due to other changes such as processing CO_2_-detection information in downstream circuitry in the brain.

Since the CO_2_-pathway is the strongest known repellency pathway for some *Drosophila* species, we wondered whether other odorants that activate the CO_2_ receptors would serve as practical repellents that can be applied easily in areas to be protected unlike CO_2_, which is expensive and impractical for use. In a previous study we had identified pyridine, an animal skin odorant, as one of the strongest activators of the CO_2_ receptor^14^. At a 10^−2^ concentration, pyridine elicited avoidance behavior in *D. melanogaster* and *D. yakuba* as expected from the response to carbon dioxide observed, and also in *D. pseudoobscura* which avoids CO_2_ to a lesser degree (Fig. 2D). In *D. pseudoobscura,* it is conceivable that other olfactory receptors may also contribute to pyridine repellency. Also, as expected the *D. suzukii* and *D. virilis* showed very little repellency to pyridine. A lower concentration of pyridine (at 10^−4^) was also tested; however, none of the species showed behavioral response in the T-maze Assay (data not shown).

The T-maze assays the instantaneous behavioral response of walking flies offered a choice between an odor and control. In order to test behavioral response of flying *Drosophila* in a long-term behavior we utilized a Two-Choice Trap Assay. Ten male and ten female starved flies are placed in a cylindrical chamber containing two entry traps lured with ACV, one of which also contained the CO_2_-neuron activator pyridine as a convenient substitute for CO_2_. Both *D. suzukii* and *D. melanogaster* showed no significant avoidance of the pyridine-treated trap (Supplemental Fig. S2) (P = 0.86). While behavioral responses of free flying *Drosophila* to CO_2_ have not been tested, tethered *D. melanogaster* that can beat their wings do not demonstrate clear anti-tracking behavior to CO_2_^15^. This taken together with our results suggest that CO_2_-receptor activating odorants such as pyridine are unlikely to act as broad-spectrum repellents for *Drosophila* species, particularly for the agriculturally important pest *D. suzukii.*

### Conservation of other olfactory avoidance pathways

**Figure 3:**
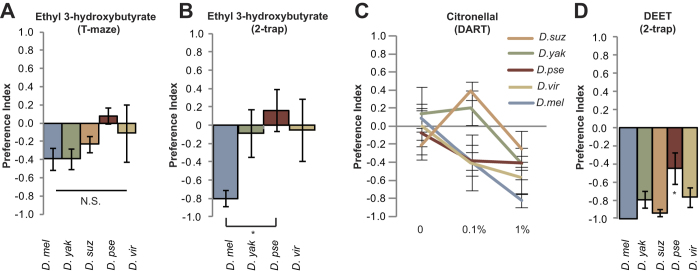
DEET avoidance better conserved across species than two other repellents carriage return. Mean preference index of *Drosophila* species to 1% ethyl 3-hydroxybutyrate in **(A)** T-maze Assay. N = 5–6 trials, 40 flies/trial, error bars = S.E.M. For Kruskal-Wallis One Way Analysis of Variance on Ranks there is no difference between the different species (P = 0.175) **(B**) Two-Choice Trap Assay. N=6–7 trials, 20 flies/trial. Error bars = S.E.M. For Kruskal-Wallis One Way Analysis of Variance (P = 0.043), *P < 0.05 Dunn’s Test. **(C)** Mean preference index of *Drosophila* species to citronellal in the DART-assay. N = 4–8 trials, ~100 flies/trial. Error bars = S.E.M. **(D)** Mean preference index of *Drosophila* species to 10% DEET in Two Choice Trap Assay in a plate. N = 8–10 trials, ~10 female flies/trial. Error bars = S.E.M. Kruskal-Wallis One Way Analysis of Variance (P = 0.023), specifically pairwise multiple comparison using Dunn’s Method showed *D. melanogaster* and *D. pseudoobscura* response is significantly different (*P < 0.05).

These findings prompted a systematic investigation of known repellent olfactory pathways in *D. melanogaster* and their conservation in related species. A second avoidance pathway in *D. melanogaster* is mediated by activation of Or85a, a member of another receptor gene family^16^. The strongest known activator of this receptor (identified by electrophysiology) is the odorant ethyl 3-hydroxybutyrate^17^,^18^,^19^. We tested two concentrations of ethyl 3-hydroxybutyrate (10^−2^ and 10^−4^) in a T-maze Assay. All species showed no preference at the lower concentration. *D. melanogaster* had some avoidance of ethyl 3-hydroxybutyrate at the higher concentration (Fig. 3A). This response was conserved in *D. yakuba.* The *D. suzukii* showed a small repellency; however, the distantly related species *D. virilis* and *D. pseudoobscura* showed no behavioral response to ethyl 3-hydroxybutryate. These behaviors are consistent with the observation that *D. pseudoobscura* and *D. virilis* lack a functional copy of the *Or85a* genes^5^,^20^,^21^,^22^.

In order to test whether ethyl 3-hydroxybutryate can reduce attraction towards an attractive odor source and to act over a longer time period, we used a Two-Choice Trap Assay. *D. melanogaster* avoids the apple cider vinegar trap with 1% ethyl 3-hydroxybutyrate (Fig. 3B). Participation in the Two-Choice Trap Assay for *D. suzukii* was very low and was not included in this analysis. The odorant was unable to repel the three other species from the lure. These results reinforce our view that for some odorants the avoidance is not conserved, because the receptor gene is not present in the genome. In addition, ethyl 3-hydroxybutryate may not activate other repellent receptors.

A third known repellent is citronellal, a naturally occurring essential oil found in multiple plant species. Citronellal activates olfactory neurons in the antenna named ab11A and ab12A in *D. melanogaster* and a Trp channel (TRPA1) is believed to play a role in citronellal’s activation of ab11A but not ab12A^23^. The odorant receptors in these neurons are unknown. In order to test the conservation in repellency to citronellal we used the previously described Direct Airborne Repellant Assay (DART)^23^. For this non-contact assay, odorant is placed on filter paper at the bottom of a standard 15 ml culture tube, with a mesh screen placed 0.5 ml from the bottom to prevent flies from contacting the filter paper with odorant. Two tubes are placed together to form a long tunnel in which ~100 flies are given 30 minutes to choose the odorant or solvent end of the tube. We found that 1% citronellal is avoided by *D. melanogaster, D. pseudoobscura* and *D. virilis* but not as strongly by *D. yakuba* and *D. suzukii*. In fact, a lower concentration (0.1%) of citronellal was slightly attractive for *D. yakuba* and *D. suzukii* (Fig. 3C). These results suggest that citronellal is unlikely to be useful as a strong repellent for *D. suzukii.*

A recently identified repellent pathway is dependent on the olfactory ionotropic receptor, Ir40a, which is expressed in the sacculus of the antenna and detects the commonly used insect repellent DEET^24^. DEET is also detected by bitter neurons in the gustatory system^25^. In *Culex quinquefasciatus* mosquitoes, DEET has been proposed to act via odorant receptor CquiOR136; however, none of the *Drosophila* species tested here have an ortholog of *CqiOR136*^26^. To examine the response to DEET in other *Drosophilids*, the Two-Choice Trap Assay in a plate is typically used^27^. Briefly, 10 female flies are placed inside a Petri dish containing two food odor-lured traps. In order to access the food, flies must crawl over a piece of filter paper impregnated with test compound. Four species strongly avoided DEET and *D. pseudoobscura* also avoided it, but to a lesser degree (P = 0.023) suggesting that this pathway is highly conserved, unlike the other repellent pathways tested (Fig. 3D). The Ir40a protein coding sequences are also well conserved compared to conservation in the *Or* gene family across the species *D. yakuba* (88%), *D. suzukii*^33^ (70%), *D. pseudoobscura* (70%), and *D. virilis* (66%) but not as conserved as *Gr21a* and *Gr63a*. In fact, DEET repellency is conserved across most tested insects, as is *Ir40a*^28^.

### Safer DEET substitutes repel an agricultural pest, the spotted wing Drosophila

**Figure 4:**
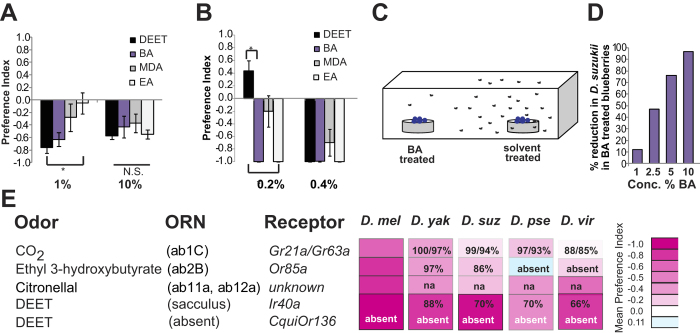
Identifying an effective natural repellent for spotted wing *Drosophila* **(A)** Mean Preference Index of *D. suzukii* to 1% and 10% for DEET, BA, MDA and EA in a 48 hour Two-Choice Trap Assay in a plate. N = 4–8 trials, ~10 female flies/trial. Error bars = S.E.M. Kruskal-Wallis One Way Analysis of Variance on Ranks for 1% DEET (P=0.027) and for 10% DEET (P = 0.926). Dunn’s test *(P < 0.05). **(B)** Mean preference index of *Drosophila melanogaster* to 0.2% and 0.4% for DEET, butyl anthranilate (BA), methyl N,N-dimethylanthranilate (MDA) and ethyl anthranilate (EA) in Two-Choice Oviposition Assay with grape juice plates. N = 5–10 trials, ~25 female flies and 15 male flies/trial. Error bars=S.E.M. , Kruskal-Wallis One Way Analysis of Variance on Ranks for 0.2% odorants is (P =<0.001) and for 0.4% odorants (P = 0.004), Tukey Test (*P < 0.05). **(C)** Schematic of the Two -Choice Assay with blueberries. **(D)** Percent reduction of *D. suzukii* offspring (eggs, larvae and pupae) laid on BA coated blueberries vs. solvent coated blueberries after 1 week of a free access to ~30 flies/ trial in a glass chamber. Total number of eggs, larvae, pupae and adults were counted 6 days after assay end. **(E)** Table summarizing amino acid identity of repellent receptor where available and avoidance behavior across various species. Heat map intensity of magenta shading corresponds to preference index in flies. Cyan shading corresponds to positive preference index.

Recently, a number of new naturally occurring repellents were discovered that can substitute for DEET and are strongly repellent to *D. melanogaster* and mosquitoes^24^. Many of these repellent compounds are present naturally in fruits, have very mild and pleasant odors, are commonly used flavor and fragrance components belonging to a category called generally recognized as safe (GRAS) and are approved for human consumption through addition to food. We tested whether these compounds can be used to repel *D. suzukii.* We measured behavioral responses to three of these DEET-substitute compounds: (1) butyl anthranilate (BA), (2) methyl N,N-dimethylanthranilate (MDA) and (3) ethyl anthranilate (EA) in the previously used Two-Choice Trap Assay in a plate. *D. suzukii* avoided the traps containing 10% of all three compounds (P = 0.926); however, at 1%, ethyl anthranilate did not repel *D. suzukii* (P < 0.05) (Fig. 4A).

We then asked if DEET-like compounds would also act as oviposition deterrents by testing preference for *Drosophila melanogaster* egg-laying using a Two-Choice Oviposition Assay (Fig. 4B). Briefly, 15 male and 25 female flies are released into a 10 gallon closed glass chamber containing two (one with test odorant the other with solvent) Petri dishes with standard grape juice media. After 24 hours, eggs laid on grape media with test odorant and solvent are counted. *D. melanogaster* did not oviposit on grape media infused with 0.4% DEET (Fig. 4B). At the lower concentration of 0.2% DEET, there is no avoidance and even initially a preference for ovipositing on the DEET containing media. *D. melanogaster* avoided ovipositing on MDA, BA or EA at both concentrations tested. Since these DEET-like compounds deter attraction and oviposition, we wondered if they would also deter *D. suzukii* oviposition on fruit.

In order to test whether BA can protect fruit from *D. suzukii* we revised the previous assay by replacing the grape juice media with two bowls of fresh, ripe blueberries (a preferred fruit of D*. suzukii*) and extending the assay time to one week. One bowl of blueberries was painted with BA and the other solvent. This Two-Choice Assay in a glass chamber (Fig. 4C) allowed us to infer egg-laying from a count of eggs, larvae and pupae emerging from each set of fruit. As expected from the time lapsed between the end of the experiment and dissection of exposed blueberries, few eggs were observed, with the exception of one of the six trials of 10% BA where 43 unhatched eggs out of 159 total *D. suzukii* that were counted. Of the unhatched eggs, 95% were laid on the control blueberries. More importantly, we found a clear dose-dependent decrease in numbers of larvae and pupae emerging from the BA treated blueberries. Remarkably, decreases were observed from the week-long experiment after only a single treatment, with substantial decreases at 2.5% and nearly complete protection at the 10% concentration (Fig. 4D). This proof of principle experiment indicates that insect repellents with different safety profiles can indeed be useful to reduce fruit damage during ripening.

## Discussion

Each year *D. suzukii* damages hundreds of millions of dollars worth of fruits worldwide and there is a great need to find new ways to reduce this loss^29^,^30^. Toxic insecticides are often risky to use directly on fruits, and a safe affordable repellent could provide protection and reduce use of toxic chemicals. Although DEET is repellent to *D. suzukii,* it is a synthetic chemical that is unlikely to be useful in protecting crops given the human health concerns regarding food supply contamination, as well as the high production cost for the large volumes that would be required in agriculture. Other insect repellents we test here provide an opportunity to develop alternative effective strategies to reduce fruit damage. More generally, insects destroy a very large fraction of the global agricultural output and necessitate millions of tons of toxic insecticide use that is environmentally unfriendly and harmful to human populations. Further analysis of possibly environmentally safer and non-toxic repellents could decrease use of such insecticides. The analysis of conserved repellent pathways in the insect olfactory system offers an avenue to design behavioral control strategies of these dangerous pests and ultimately could form a foundation of novel and safe technologies that can improve both plant and human health.

## Materials and Methods

### *Drosophila* stocks

*D. yakuba*, *D. pseudoobscura, D. virilis* and *D. melanogaster* (wild-type lines) were obtained from the San Diego Stock Center. *D. suzukii* were a generous gift of R. Stouthammer. Unless otherwise indicated *D. melanogaster* were *white*^*1118*^ backcrossed 5X to Canton-S. *D. melanogaster* wild-type A1 was caught in La Jolla, California and A2 in Point Loma, California in July 2011. These wild caught *D. melanogaster* were tested within five months of being captured. *D. melanogaster*, *D. yakuba* and *D. virilis* were raised on standard cornmeal in a humidified incubator at 25 degrees Celsius on a 12 hour light/12 hour dark cycle. *D. pseudoobscura* were raised in the dark at 18 degrees C. and *D. suzukii* were raised on a modified cornmeal diet at room temperature.

### Single-sensillum electrophysiology

 Recordings were obtained as described previously^7^.

### Short-term assays

Contact and non-contact short-term behavioral assays were conducted to determine responses to odorants.

*The T-maze Assay* is ideally suited for highly volatile compounds such as CO_2_. Avoidance to carbon dioxide and pyridine trials were conducted as before^7^. Briefly, approximately 40 flies are released from an elevator into the horizontal intersection of a T-shaped apparatus. A test odorant is applied to one arm of the T-maze and a control odorant to the opposite arm. For trials with other odorants, paraffin oil was used as the solvent and in the control arm. Flies are given one minute to choose an arm before the elevator closes. Orientation of arms for test and control were switched between trials. Preference index was calculated as = (number of flies in test arm-number of flies in control arm)/(number of flies in test arm + number of flies in control arm).

The *Direct Airborne Repellent Test (DART)* is suited for volatile compounds that could be confounded by responses from the taste system. Trials were performed using slight modifications to the previously reported assay^23^. Fifty flies were aged per vial and starved in vials with 2 Kimwipes moistened with 3 ml of water. A 6 mm diameter circle of Whatman #1 filter paper was placed in the bottom of a 10 cm length tube (VWR, #60818-661) to deliver the odor. A brass screen of 8/32 inch diameter was placed 5 mm from the bottom of the tube to gate off the filter paper. Approximately 100 flies were inserted into the control tube and joined to the tube with test odorant. After 30 minutes exposure in the dark at 25 degrees Celsius, the apparatus was photographed. Flies 5 cm from each screen were counted. Preference index was calculated.

*The Two-Choice Trap Assay* in a plate tests less volatile odorants. Trials were performed as described^27^. Ten female flies are placed in a Petri dish containing two traps. Traps were made with 1.5 ml micro centrifuge tubes (USA Scientific) with opening cut in the bottom of the tube. Both traps contain the fly’s normal laboratory food at the base. The neck of one trap has a filter paper with test odorant, the other trap has solvent. Five microliters of hexane (control) and five microliters of 10% DEET or test compounds in hexane were applied to the stem part of filter paper inserted into upper part of pipette tip near entrance to trap to allow flies to walk over treated surface. Traps were placed in chemical hood for 5 minutes to allow hexane to volatilize before being placed in the 1% agarose treated Petri dish chamber.

### Long Term Assays Carriage Return

#### Two-Choice Trap Assay

Assay was performed to determine^24^ preference for an attractive food source in the context of a repellent odor. Briefly, ten male and ten female starved (4–7 day old) flies are placed in a cylindrical chamber containing two traps: (1) with test odorant and lure, (2) with solvent and lure. Apple cider vinegar (10%) is the lure for all trials except for *D. virilis* where liquid malt (25%) was used (*D. virilis* is not attracted to apple cider vinegar^31^). To create a well for separating lure from test odorant, a single cap cut from a BioRad PCR 0.2 ml Tube Flat Cap strips (#TCS0803) was inserted in a snap top lid of a microcentrifuge tube. To run the assay, 35 ul of test odorant was pipetted into inner well and 90 ul of lure into the outer ring. For all trials, the control trap had paraffin oil solvent in inner well and lure in outer ring. Flies were given six hours to enter traps. Preference index was calculated.

#### Two-Choice Oviposition Assay

Test odorant or solvent was added to warm standard grape juice media in Petri dishes and set to solidify. Petri dishes were placed at opposite ends of a 10 gallon closed glass chamber. A 100 ml beaker containing 40 ml of distilled water was placed equidistant between grape plates to add moisture to the chamber. For each trial, 15 male and 25 female un-starved Canton-S flies were lightly anesthetized with carbon dioxide and released in the chamber. Assay was run for 24 hours at 25 degrees Celsius on a 12 hour light: 12 hour dark cycle. Preference was determined by counts of eggs on grape plate containing test odorant and control.

#### Two-Choice Blueberry Assay in a glass chamber

Fresh blueberries were obtained from a local grocer and were soaked in distilled water for 30 minutes, rinsed and dried. To prepare the chamber, 31 grams of blueberries were placed in each of 2 plastic bowls. Test compound (0.4 ml) is painted on blueberries in test bowl and solvent (0.4 ml) on blueberries in control bowl. Bowls are placed at opposite ends of a 10 gallon closed glass chamber. A 100 ml beaker containing 40 ml of distilled water was place equidistant between fruit to add moisture to the chamber. For each trial, 15 male and 15 female un-starved flies were lightly anesthetized with carbon dioxide and released in the chamber. Assay was run for seven days at 25 degrees Celsius on a 12 hour light: 12 hour dark cycle. After 7 days, each bowl was covered and set aside for an additional six days for eggs and larvae to develop after which the blueberries were dissected under microscope and number of eggs, larvae, pupae and adults were recorded. Preference was determined by inferring egg-laying from a count of eggs, larvae and pupae emerging from each set of fruit.

## Additional Information

**How to cite this article**: Krause Pham, C. and Ray, A. Conservation of Olfactory Avoidance in *Drosophila* Species and Identification of Repellents for *Drosophila suzukii*. *Sci. Rep.* **5**, 11527; doi: 10.1038/srep11527 (2015).

## Supplementary Material

Supplementary Information
