# Supplementary material for: Conservation of Olfactory Avoidance in Drosophila Species and Identification of Repellents for Drosophila suzukii
Source: Sci Rep. 2015 Jun 22;5:11527. doi: 10.1038/srep11527 (PMC4476414; doi:10.1038/srep11527)
Supplement: Supplementary Information [file srep11527-s1.pdf]

**TITLE: Conservation of Olfactory Avoidance in *Drosophila* Species and Identification of Repellents for *Drosophila suzukii***

**AUTHORS:** Christine Krause Pham[1] and Anandasankar Ray[1,2*]

[1]Interdepartmental Neuroscience Program and [2]Entomology Department, University of California, Riverside, CA 92521.

*Correspondence: Anandasankar Ray, anand.ray@ucr.edu, Ph#+1-951-827-5998.

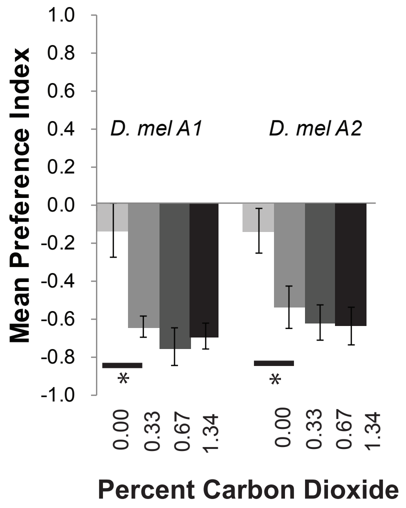


**Figure S1. Wild-type *D. melanogaster* lines recently introduced into laboratory robustly avoid $CO_2$.** Wild caught *D. melanogaster* were tested in the T-maze Assay within five months of being captured. (*D. melanogaster* wild-type A1 was caught in La Jolla, California and A2 in Point Loma, California in July 2011. *D. melanogaster* species morphological identification was confirmed by sequencing the mitochondrial cytochrome oxidase gene (COI) gene at the San Diego Stock Center). Mean preference index to $CO_2$. N= 6-8 trials, ~40 flies/trial. Error Bars= S.E.M. Two-tailed T-test between 0 and 0.33% $CO_2$ (A1, p=0.01, A2, p=0.03). * p = 0.05.

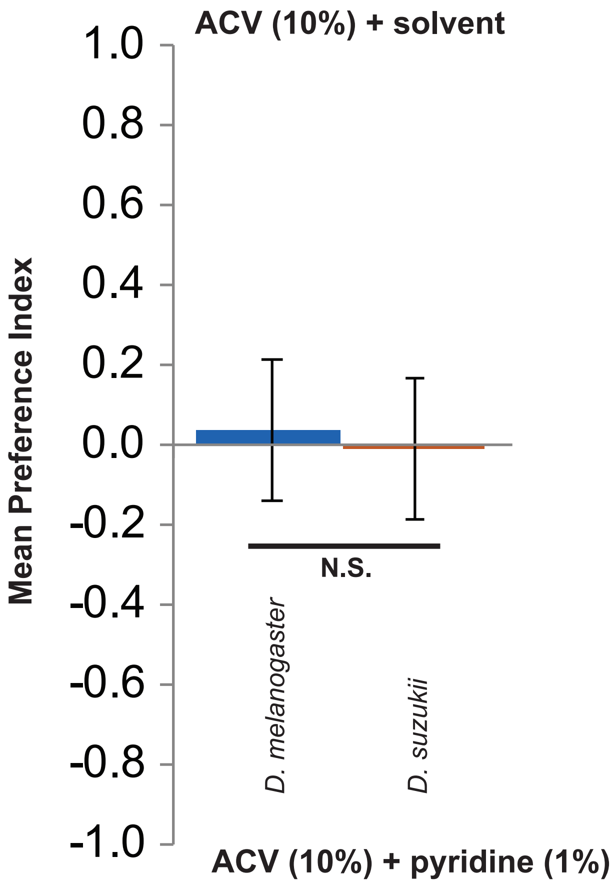


**Figure S2.** ***D. melanogaster*** **and** ***D. suzukii*** **show no avoidance to an activator of the $CO_2$ receptor in the presence of apple cider vinegar.** Two-choice Trap Assay with 1% pyridine. Flies are given 6 hours to choose between the trap containing 10% ACV and pyridine or solvent. N=5 trials, 20 flies/trial. Error bars = S.E.M., Two-tailed T-test is Not Significant (N.S.) p=0.86.
